# A Lecture on Menorrhagia, Vel Hæmorrhagia Uteri, or Uterine Hæmorrhage

**Published:** 1839-03-30

**Authors:** N. Chapman

**Affiliations:** Professor of the Theory and Practice of Physic, in the University of Pennsylvania


// MEDICAL EXAMINER.

DEVOTED TO MEDICINE, SURGERY, AND THE COLLATERAL SCIENCES.

No. 13.] PHILADELPHIA, SATURDAY, MARCH 30, 1839. [Vol. II.

### A LECTURE ON MENORRHAGIA, VEL HÆMORRHAGIA UTERI, OR UTERINE HÆMORRHAGE. *By* N. Chapman, M. D., *Professor of the Theory and Practice of Physic, in the University of Pennsylvania.*

[Reported for this Journal.]

An opinion having been formerly entertained that the whole of the extravasations of a sanguineous aspect from the womb were of a menstrual nature, the former term, which means an undue flow of the menses, was applied pretty much in the sense in which we now employ hæmorrhagia uteri, or uterine hæmorrhage. Elsewhere,* I trust I have shown, that the catamenia, instead of blood, are a peculiar fluid, the product of a secretory action of the uterus. Nor is it true, as many suppose, that all of the periodical discharges from this source are menstrual. On the contrary, I have found, in every instance in which such were copious, pure coagulable blood to be emitted. Even where, in the commencement, the fluid seemed to be partially menstrual, it lost that character, and became blood.

Granting, then, the correctness of this view, and which I think very few can now be found to deny, the impropriety of the term menorrhagia is obvious. Convinced of this, some have proposed the substitution of metrorrhagia. But as it means only a discharge from the womb, it is vague and unsatisfactory. Nothing can more precisely express the affection than hæmorrhagia uteri, and hence it should be adopted to the exclusion of all other titles.

This hæmorrhage may take place in the unimpregnated or impregnated state of the organ, and precede or succeed delivery. The latter is occasioned in a mode, namely, by the rupture of vessels, which removes it from my consideration, and will be resigned to the department of midwifery. To me it belongs to treat only of the former, as properly vital, or spontaneous hæmorrhage. This may recur monthly, with considerable exactness, or more irregularly, at shorter or longer intervals, or continue almost uninterruptedly. But the law of periodicity is observed by it with greater uniformity than by any of its kindred affections.

An attack of an active uterine hæmorrhage may be ushered in without any, or a very slight premonition, though generally by a train of precursory symptoms, lassitude and weariness of the limbs especially,—sometimes chilliness, followed by fever, or, at least, by increased force or acceleration of pulse, headache, flushed face, embarrassed respiration, a sense of fulness in the uterus, pain, acute or dull, in the lumbar region, or groins, with sensations of dragging or bearing down, attended by a frequent desire to urinate, and occasionally by tenesmus. These phenomena are often connected with much of that sort of feeling, expressed by the vague term nervousness. The discharge appearing, not a little relief is afforded, unless it be very profuse, when the antecedent suffering is exchanged for the wretchedness of exhaustion, sometimes with nausea or vomiting, coldness and shivering, disposition to syncope, &c. &c.

Among the remote causes of uterine hæmorrhage, the most conspicuous is the period of life. It is seldom met with previously to the season of puberty,—is very apt to occur slightly, in anticipation of the complete establishment of the menses,—again, when they are about to cease, and, sometimes, very copiously. No period, however, between these extreme points, is exempt from attacks.

The predisposition is also dependent on certain constitutional states, and the character of the hæmorrhage modified accordingly,—the active variety being chiefly found in the sanguineous, the florid, and robust,—and the less active, or passive, in the enervated, relaxed, and phlegmatic.

More particularly is blood directed to the womb, by the habits of sitting, or luxurious indolence, or such employments or amusements as spinning, or dancing, or equitation, or walking rapidly, in which the lower extremities are exerted, or by excess of venery, or the reverse, abstinence from it, where the desire is urgent, or by numerous labours, or repeated abortions, or by the prevalence of leucorrhœa, constipation, or frequent purging, with articles operating mainly on the rectum, and through it on the uterus,—certain emmenagogues, an undue use of the warm bath, or of foot stoves, &c.

Besides these ordinary agencies, hæmorrhage is sometimes the consequence of a series of organic lesions of the uterus,—induration or softening of texture, common ulceration, scirrhosity or open cancer, polypi, and various tumours,—fungoid, or other morbid growths, of diverse sorts. Connected, however, with such states, the effusion, I suspect, proceeds mostly from rupture of vessels, and hence does not strictly appertain to the present inquiry.

No difficulty can exist in distinguishing the uterine from other hæmorrhages. Menorrhagia is most apt to be confounded with it. An inspection of the discharge will, however, at once, remove all doubts, it being in the one pure coagulable blood, and in the other, a thin, dark fluid, of a peculiar odour. Between the blood, in some of the less active hæmorrhages, and the menses, there is a closer resemblance, and great attention will be required in the discrimination. Embar-

* Elements of Therapeutics and Materia Medica.

rassment, too, may be experienced in this respect, as relates to the hæmorrhage dependent on those structural derangements of the womb, just mentioned, though here, the obscurity is cleared away by an examination *per vaginam.*

It seldom happens that there is any immediate danger in the active form of this hæmorrhage, provided it be spontaneous, to whatever extent it proceeds. Death, at least, rarely, or perhaps never, suddenly ensues from it. Much, however, is to be apprehended in the ulterior consequences, where it is frequently repeated or long continued, by the constitutional disturbance, and general derangement of health, of which it is productive. But it is otherwise in the less active, or passive states of the disease, the loss of blood here being sometimes most copious, and the effects truly alarming. I have met with some instances, and many are reported, where pints of blood have escaped in an inconceivably short time,—and though never within my own observation ending fatally, such events have undoubtedly taken place. Yet it would seem, that from no part of the body is excessive hæmorrhage better borne than the uterus, or the preservation of life more frequent, under apparently desperate circumstances.

Commonly, the active hæmorrhage is easy of cure. The cases which prove intractable are of long standing, to be found, for the most part, in women somewhat advanced in life, very often about the season of the cessation of the menses, with some organic lesion of the uterus, or of a lymphatic temperament, and general bad condition of system.

The rareness of a fatal termination in either state of this affection, has prevented the acquisition of any precise knowledge of its anatomical characters. No doubt, however, they are the same as in hæmorrhages of other mucous surfaces, and which have been sufficiently detailed under some preceding heads. The organic lesions, I have said, seldom bear a relation to vital hæmorrhage, and hence, need not be again enumerated, or more minutely described.

With those who confess the peculiar obscurity in which the pathology of uterine hæmorrhage is involved, I cannot unite. To me it is as plain as that of any of its affiliated affections. Lined as the womb is, with a mucous membrane, why should it not be subject to hæmorrhage? But the chief difficulty complained of seems to relate to those cases where pure blood periodically escapes, instead of real menses, or in which the two fluids are mixed. Every hæmorrhage displays such a tendency occasionally,—and that it should be more strikingly manifested in the uterine, is readily to be conceived, from the natural functions of the organ. The uterine vessels, I have said on a former occasion, when in a healthy state, by virtue of their secretory office, periodically exercised, convert the blood into a peculiar fluid. Disordered, however, they lose this capacity, and blood is exhaled more or less unchanged. But it is asked, whether different sets of vessels are not engaged in these operations? Recurring to what was delivered on the general pathology of hæmorrhage, an explanation of this phenomenon will be acquired. No more of that discussion shall I now recapitulate, than merely the remark, that the secretory vessels of a part may be so differently influenced at the time, that while one portion of them is adequately performing its duty, another shall allow blood to pass through them, little or not at all affected. There is not the slightest necessity to suppose a double set of vessels, to solve the problem.

In a well-marked case of the active state of this hæmorrhage, we have to contend with a plethoric, and perhaps an inflammatory condition, local or general. Excepting, from the inconvenience of it, I am not aware of any objection to permitting it to continue as an effort of nature, in most instances, at least, to get clear of a morbid irritation, or redundant blood. Deeming it, however, expedient on any account, to interfere, we shall have little doubt as to the remedies. To a considerable extent, venesection becomes necessary,—the bowels, when constipated, should be opened by a mild laxative, and the nitrate of potash, with a modicum of tartarized antimony, subsequently directed. The latter is undoubtedly among the most suitable of our remedies at this time.

Digitalis has been proposed here, even as a substitute for venesection,—and we shall find it most strenuously urged with this view, especially by Currie, Ferriar, and Drake. No substitute for it, however, have we in this, or in any other instance, of a plethoric or active circulation. Digitalis, in this case, is to be placed on nearly the same footing with some other sedatives. The pulse being without force or volume, though quick and irritated, it may be recurred to, provided the hæmorrhage be not copious, since it is apt to induce a relaxed or patulous state of the vessels, and hence to increase the flow of blood. Nevertheless, under such circumstances, or where venesection is no longer admissible, the most appropriate means, usually, is cupping the lumbar region, or an application of a blister to the same part, or both, in succession.

Certain astringents are next resorted to, and, properly directed, may be serviceable, though I have some doubts of their efficacy. Nothing, however, is better established, than that these articles ought to be preceded by depletion. Let this be omitted, or too timidly employed, and these and all other means will prove inefficient, and sometimes positively detrimental.

Of the class of astringents, the acetate of lead stands probably first in repute, and is, indeed, represented as sometimes displaying extraordinary powers. Many, as well of Europe as this country, who have used it largely, concur in this estimate of its value. Heberden says, "if ever there was a specific in any disease, it is surely the lead in uterine hæmorrhage." The late Professor Barton was equally lavish of his commendations of it. My own experience will not allow me to go so far, and I even suspect that the accounts of its efficacy are great exaggerations. It is prescribed in the dose of two or three grains, with a quarter of a grain, or less, of opium, to be repeated at shorter or longer intervals, according to the emergency.

As the other articles of this description are more employed in the less active state of this hæmorrhage, I shall postpone my animadversions on them till I come to that portion of the subject, and now turn to the consideration of a very different set of medicines. The first which attracts notice is ipecacuanha.

No inconsiderable testimony might be adduced to its powers, which, in my opinion, are superior to those of the acetate of lead. The mode of giving it is in minute doses, with a small portion of opium, so as scarcely to distress the stomach, under an apprehension of exciting vomiting. What would be the effect of this process induced by an emetic, I cannot say from any knowledge of my own in active uterine hæmorrhage. Cases of it, however, I have seen to cease on the coming on of spontaneous vomiting,—and I can discern no objection to the use of emetics, provided there has been previously a reduction of vascular action. But of this again, presently.

More effectual than either of these articles is the ergot, according to recent declarations of some practitioners. That it controlled the floodings preceding and following delivery, had been long known,—and it was presumed that it would here prove still more decisive. Not positively denying its utility, for I have very slight experience with it, I do still think, that the analogical reasoning which led to the extension of this application of it, is incorrect. The hæmorrhage in the two cases is produced very differently, and seems to require remedies equally dissimilar,—in the first, by the rupture of vessels, to be compressed by uterine contractions, and in the second, by a mere exhalation of blood, which is checked by an alteration in the state of the capillaries. Be this, however, as it may, we are told that the tincture of ergot answers far better than the powder. The dose is thirty or forty drops, occasionally repeated.

Evidence of a very respectable character might be collected, of the utility of opium, and under various circumstances of the hæmorrhage. Yet I cannot help thinking, that it has been abused by a too general and indiscriminate application of it. Thus it appears to me, that, prescribed in a full dose in the early stage of active hæmorrhage, under ordinary circumstances, its effects would be injurious. We, however, meet with such cases attended by pain, irritation, and spasm of the uterus, by which irregular movements the effusion of blood is excited or kept up. Here, after sufficient bleeding, opium signalizes its utility, acting on a principle too plain to require any explication. Nor am I prepared to limit its application exclusively to this condition. The more I prescribe it, the stronger is my conviction that it exercises a very general power over hæmorrhage, provided adequate depletion has been practised, and which I think it does by its operation on the nervous system, as formerly explained. Commonly, it, or some one of its preparations, is given alone,—but the Dover's powder often answers better,—and, in some instances, a union of opium, ipecacuanha, and camphor, is still more to be preferred.

The most prominent of the general remedies, with which we endeavour to arrest uterine hæmorrhage, of the species under review, have been mentioned. There are, however, some topical expedients,—among which is an application of cloths wrung out of cold water, or vinegar, or ice itself, to the pudendum, or to pour down water from a height in a small stream on the belly.

An advantage, we are told, may also be gained by injecting into the vagina a solution of alum, sugar of lead, white vitriol, or other astringent fluids. The rectum is resorted to by some, as a medium of administration of these articles, it being affirmed that they thus act with greater efficacy. Whether it be so, I am unable to state from any trials of my own. Yet where much irritation and spasmodic action of the uterus prevailed, I have sometimes derived great benefit from opiate enemata. More, however, to be trusted than any of the means I have suggested, is plugging up the vagina, so as to allow a coagulum of blood to form,—and the best substance for the purpose is sponge; though tow, flax, cotton, or even soft rag, may be substituted.

(To be continued.)

